# Announcement: Fifth International Symposium on Metal Ions in Biology and Medicine

**DOI:** 10.1155/MBD.1997.289

**Published:** 1997


					FIFTH INTERNATIONAL SYMPOSIUM
ON METAL IONS IN BIOLOGY AND MEDICINE
8-10 May 1998
Neuherberg/Munich, Germany
http://www.hmi.de/bereiche/N/NG/Events.html
The "Fifth International Symposium on Metal Ions in Biology and Medicine" will be held in
Neuherberg (Munich), Germany, from 8 to 10 May 1998, as a cooperation between the Hahn-
Meitner-lnstitut Berlin, Department of Trace Elements in Health and Nutrition and the GSF-National
Research Centre for Environment and Health, Institute for Ecological Chemistry, Neuherberg,
Germany. This symposium will be aimed to guarantee the free and effective exchange of opinions
between specialists working in analysis, research and applications of Metals and Trace Elements in
areas of biochemical, biological and medical sciences. It will promote an intensive and productive
dialogue between these groups of experts, regarding future collaboration. The scientific program will
include plenary and keynote lectures, plattform sessions, posters with discussions and debates.
The first symposium was held in 1990 in Reims, France (chairmen: Ph. Collery and J. C. Etienne)
and has been continued every two years: 1992: Loutraki, Greece (chairmen: J. Anastassoupoulou
and Th. Theophanides); 1994: Montreal, Canada (chairmen: N. A. Littlefield and L. Poirier); 1996:
Barcelona, Spain (chairmen: J. L. Domingo, J. Corbella, J. M. Llobet and Ph. Collery).
The Scientific Committee decided to focus the "Fifth International Symposium on Metal Ions in
Biology and Medicine" on the following aspects:
Metals and Environmental Health
Speciation of Metals and Other Elements
Uses of Metals and Selenium in Clinical Applications
Metals and Ageing
Metals and Homeostasis
Effect of Low and High Nutritional Trace Element Intake
Metals and Other Elements Interactions
Assessments of Trace Element Status and Health
Toxicity of Metals
Metals and Hormone Receptions
Metals and Chelation Therapy
Metals and Enzyme Activity
Advanced Methods of Analysis
Language: English
Location: GSF-Forschungszentrum, Neuherberg (15 minutes from Munich, Germany)
Registration fee:
500 DM full (participants), 270 DM (students) (lectures access, final program, proceedings, welcome
reception, symposium-dinner and coffee-breaks included)
Reduced rates:
200 DM (one day participants: only coffee-breaks included)
270 DM (retired scientists: welcome reception and coffee-breaks included)
125 DM (accompanying persons: welcome reception, coffee-breaks and conference dinner
included)
Special rates:
10% reduction for members of the FESTEM
450 DM (full participants for registration before March 1st 1998)
800 DM (registration before March 1st 1998, for participants of both conferences: the pre-
conference "First-Speciation Conference: Trace Element Speciation in Biomedical, Nutritional and
Environmental Sciences" [held from 4 to 7 May 1998 also in the GSF-Forschungszentrum,
Neuherberg: for more information: Congress service, Postfach 1129, D-85758 Oberschleil3heim,
Germany, Fax: + 49 089 3187-3362, e-mail: schroedel@gsf.de] and the "Fifth International
Symposium on Metal Ions in Biology and Medicine"
Proceedings: the congress proceedings will be published by John Libbey Eurotext Publishers and
indexed in the principal database, including Current Contents
289
Limited number of participants: 300
Preliminary program
Thursday 7 May 1998
Pre-registration at the Hotel Ibis. Welcome reception
Friday 8 May 1998
Session I: Metals and Homeostasis
R. J. P. Williams, UK, Homeostasis, transport and cellular mechanism of metals
Session I1: Toxicity of Metals
F. W. Sunderman, Jr., USA, Teratogenicity and embryotoxicity of metals in humans
Session II1: Metals and Enzyme Activity
I. Bremner, UK, Role of metallothionein in cellular metabolism
Session IV: Speciation of Metals and Other Elements
S. Fairweather-Tait, UK, Dependence of the availability on the metal species
Poster session (with snacks and drinks)
Saturday 9 May 1998
Sessions V and Vl: Metals in clinical applications
D. Templeton, Canada, Progress in the safe chelation of iron
R. Cornelis, Belgium, On the importance of the pre-analytical steps in clinical uses of trace element
analysis
Sessions VII and VIII: Metals in Clinical Applications
P. Chappuis, France, Copper related-diseases
G. Vivoli, Italy, Metals and chronic degeneration diseases
Poster session II (with snacks and drinks)
Congress dinner
Sunday 10 May 1998
Session IX: Clinical Aspects of Selenium-intake
J. T. Salonen, Finland, The role of Hg and Se, and Hg and Fe in lipid peroxidation and human
atheriosclerosis
G. F. Combs, Jr., USA, Clinical efficacy of selenium supplements in cancer prevention
J. Neve, Belgium, Bioavailability and safety of selenium supplements
J. Vanderplas, Belgium, Human selenium deficiency and thyroid clinical status
H.-J. Gramm, Germany, Selenium and selenoprotein during the acute inflammatory response. Is
there an indication for Se-supplementation in severe sepsis?
Round table (Chair: G. F. Combs, Jr.)
Instructions for the abstracts available from
Dr. Virginia Negretti de Br&tter, Organizing Committee
Fifth International Symposium on Metal Ions in Biology and Medicine
Hahn-Meitner-lnstitut Berlin
Glienicker Str. 100
D-14109 Berlin, Germany
fax: + 49 030 8062 2262 or 2781
e-mail: negretti @ hmi.de
Deadline for the submission of manuscripts: 2nd March 1998.
The proceedings will be ready at the begin of the symposium.
Registration:
Mrs. Ulla SchrSdel
GSF-Forschungszentrum, Neuherberg, Ingolst&dtler Landstral3e 1,
D-85764 Oberschleil3heim, Germany, Fax: + 49 089 3187-3362, e-mail: schroedel@gsf.de)
only for registration
Registration form available under
http://www,hmi.de/bereiche/N/NG/Events,html
290

				

